# Ultrasound Measurement of Femoral Articular Cartilage Thickness Before and After Marathon Running

**DOI:** 10.7759/cureus.52870

**Published:** 2024-01-24

**Authors:** Matthew K Lunser, Mark Friedrich Hurdle, Walter C Taylor, Raphael A. O Bertasi, Tais G. O Bertasi, Svetlana Kurklinsky, George M Cooper, Hillary W Garner, Haytham Helmi, George G. A Pujalte

**Affiliations:** 1 Department of Family Medicine, Mayo Clinic, Jacksonville, USA; 2 Department of Pain Management, Mayo Clinic, Jacksonville, USA; 3 Department of Internal Medicine, Mount Sinai Morningside West, New York, USA; 4 Department of Radiology, Mayo Clinic, Jacksonville, USA; 5 Department of Critical Care Medicine, Mayo Clinic, Jacksonville, USA

**Keywords:** knee ultrasound, long-distance running, diagnostic musculoskeletal ultrasound, articular cartilage, sport biomechanics

## Abstract

Objective: The purpose of this study was to use ultrasonography to measure femoral articular cartilage thickness changes during marathon running, which could support MRI studies showing that deformation of knee cartilage during long-distance running is no greater than that for other weight-bearing activities.

Materials and methods: Participants included 38 marathon runners with no knee pain or history of knee injury, aged 18-39. Ultrasound images of the femoral articular cartilage were taken two hours before and immediately after the race. Femoral articular cartilage thickness was measured at both the medial and lateral femoral condyles.

Results: The maximum change in femoral articular cartilage thickness, measured at the left outer lateral femoral condyle, was 6.94% (P=.006). All other femoral articular cartilage thickness changes were not significant.

Conclusion: A change in femoral articular cartilage thickness of 6.94% supports our hypothesis that long-distance running does not induce deformational changes greater than that of regular daily activities. This study using ultrasonography supports MRI evidence that knee cartilage tolerates marathon running well.

## Introduction

The articular cartilage is a dynamic tissue capable of undergoing deformation and subsequent recovery [1-10]. It can be injured as a result of mechanical overload from an acute stress or by progressive overload [11-14]. Injury to the articular cartilage can lead to degenerative joint disease, which is a costly worldwide burden [15]. The knee is commonly affected by degenerative joint disease, particularly at the femoral articular cartilage (FAC) in the early stages of disease [16].

Deformational changes to FAC are affected by the amount of stress and type of activity [2,3,7]. The FAC undergoes a normal diurnal thickness change, with focal areas becoming 4-10% thinner in the afternoon from normal daily weight-bearing activities [17-19]. Although running generates forces up to five times body weight on the lower extremities [20], running for up to one hour has been shown to produce deformation in FAC similar to that resulting from activities of daily living (i.e., 3-5.3% decrease in cartilage volume) [21,22]. By this measure of FAC function, the knee appears to tolerate running moderate distances well, which is consistent with evidence that short- to moderate-distance running does not cause degenerative joint disease [23]. As longer-distance races have become more popular, the question of a safe upper limit to running and the ability of cartilage to withstand increased cumulative stress becomes important.

Magnetic resonance imaging (MRI) has been used to study changes in knee cartilage before and after marathon running [24-28]; however, to our knowledge, FAC thickness changes have not been reported on ultrasound for marathon running. Ultrasound imaging can be done with portable equipment, is less costly than MRI, and has been used to reliably assess [29-33] and measure the thickness of the FAC [34-38] in a number of studies [39-42]. The purpose of this study was to evaluate the deformation of a weight-bearing portion of the FAC using ultrasound before and after marathon running. We hypothesized that the deformation change after a marathon is not significantly different than the deformation change that has been reported for other less demanding activities.

## Materials and methods

This prospective cohort study was approved by the Institutional Review Board of Mayo Clinic (approval number: 14-008627), and oral informed consent was obtained before participants were enrolled. Written consent was waived for this minimal risk study.

Patients

This prospective study took place after a marathon race on February 15, 2015. Participants were included if they did not have a history of knee pain or knee injury, while they were excluded if they had a history of injury to the anterior cruciate ligament, FAC, or meniscus; knee pain that had limited running during the six months before the start date of the study, February 15, 2015; or a body mass index greater than 30 kg/m^2^.

Ultrasonography

Two ultrasonography machines (GE Logiq, GE Healthcare, Chicago, Illinois, United States) with linear array probes were used for the study. Machines were set to B-mode at 12 MHz and a depth of 3 cm. The ultrasonography examination was performed by two investigators, one with 10 years of experience and one with six months of experience. Baseline ultrasound images of the FAC were obtained in the morning within two hours before the start of the marathon. Images were also obtained as soon as possible at the finish line after the participant finished the race.

Images of both knees were obtained in the full-knee flexion view, as in usual practice. Participants were positioned supine on the examination table, with the foot in contact with the table. The ultrasound probe was positioned just proximal to the superior margin of the patella. The probe was oriented in the transverse plane, perpendicular to the long axis of the femur. To sharpen the articular border, allowance was made for a slight cephalad and caudal tilt of the ultrasound probe (no greater than 10° angle).

**Figure 1:**
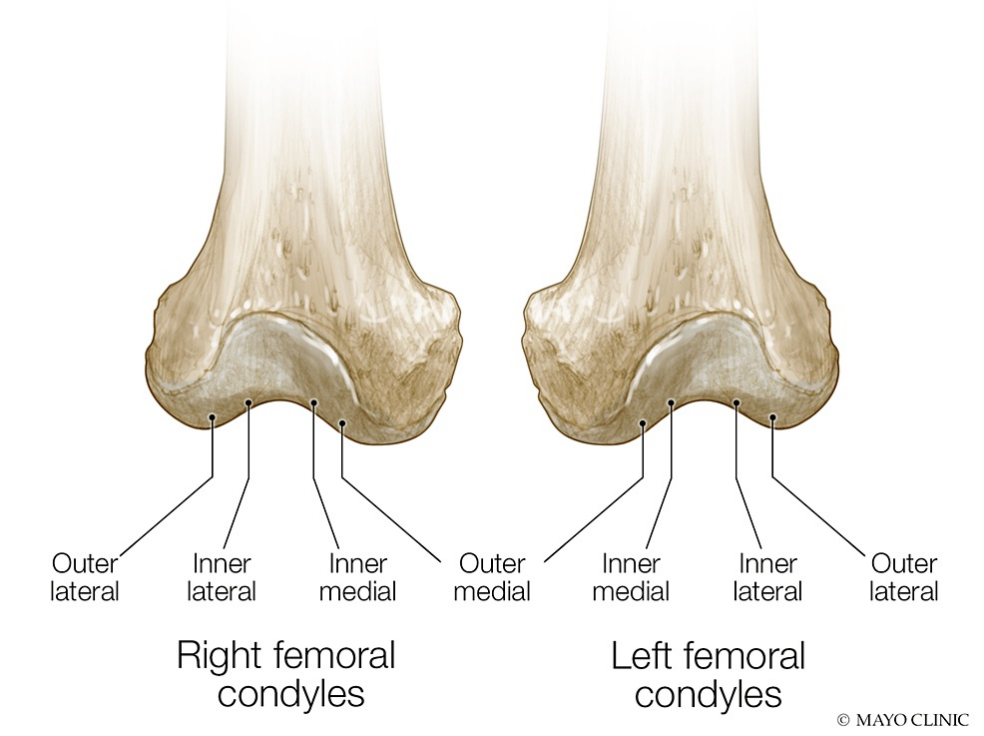
Areas of measurement for inner and outer femoral condyles Used with permission from: Mayo Foundation for Medical Education and Research

Images were measured by a single examiner using the caliper tool on the ultrasound machine. Two measurements were taken of weight-bearing portion of both the medial femoral condyle corresponding to one-third and two-thirds of the distance from the deepest portion of the intercondylar area to the peak of each condyle. Two measurements using the same method were taken of the weight-bearing portion of the lateral femoral condyle. The points closest to the intercondylar area were defined as inner, and the points closest to the peak of the condyle were defined as outer (Figure 1).

Statistical analysis

Paired t tests were used to compare outcomes of FAC thickness for the cohort before and after the race. Statistical analyses were performed using JMP software, Version 10.0.0 (SAS Institute Inc., Cary, North Carolina, United States). The results were considered significant at P<.05.

## Results

The characteristics of the study participants are given in Table 1.

**Table 1:** Participant characteristics SD: standard deviation; y: years; min: minutes

Characteristic	Mean	Range	SD
Age, y	29	18-39	5.9
Body mass index, kg/m^2^	23.8	20.05-29.02	2.4
Years of running	7.7	1-25	5.6
Miles run per week	25	8-50	9.7
Prior marathons	3.2	0-21	5.6
Time to post-race imaging, min	18	3-102	15

Thirty-eight runners aged 18-39 completed the study. The mean±SD age of the runners (17 men and 21 women) was 29±5.9 years, and the body mass index was 23.8±2.4 kg/m^2^. Prior running experience ranged from 1 to 25 years (7.7±5.6 years). Participants had completed 0-21 prior marathons (3.2±5.6). They ran 25±9.7 miles weekly. Images after the marathon were obtained at 18±15 minutes and included two participants who arrived for imaging 102 minutes after finishing the race.

Before and after changes in FAC thickness are shown in Table 2.

**Table 2:** Femoral articular cartilage thickness before and after the marathon LFC: lateral femoral condyle; MFC: medial femoral condyle

Condyle area		Right					Left				
		Mean, mm		Thickness change	Percent change	P-value	Mean, mm		Thickness change	Percent change	P-value
		Before	After				Before	After			
LFC	Outer	2	1.97	−0.0026	−1.32	.50	2.08	1.94	−0.0145	−6.94	.006
LFC	Inner	2.25	2.19	−0.0063	−2.81	.26	2.25	2.20	−0.0053	−2.34	.43
MFC	Inner	2.33	2.34	0.0005	0.23	.92	2.24	2.23	−0.0008	−0.35	.89
MFC	Outer	2.27	2.27	0.0003	0.12	.96	2.25	2.18	−0.0068	−0.31	.25

The mean FAC thickness before the race was between 2 mm and 2.33 mm for all points measured. The mean percent change for FAC thickness of the right lateral femoral condyle (LFC) after the race was −1.32% at the outer portion and −2.81% at the inner portion. The mean percent change for FAC thickness of the right medial femoral condyle (MFC) was 0.23% at the inner portion and 0.12% at the outer portion. The largest change was −6.94% at the outer portion of the left LFC; the inner LFC portion changed −2.34%. The left MFC changed −0.35% at the inner portion and −0.31% at the outer portion.

**Figure 2:**
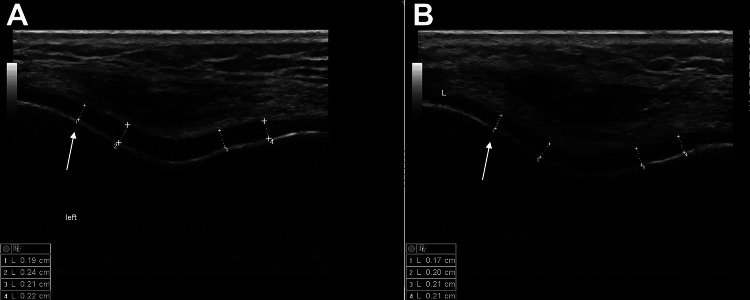
Ultrasound measurements A: Before the marathon. B: After the marathon. A significant decrease in femoral articular cartilage thickness can be seen at the left lateral femoral condyle (arrow).

The only significant change occurred at the outer portion of the left LFC (−0.0145 mm; P=.006) (Figure 2).

## Discussion

The results of the study suggest that FAC deformation that occurs with marathon running is no greater than that previously reported for other weight-bearing activities [10,17-19,21,22,43]. In the current study, the maximum deformation was a 6.94% decrease in thickness in the outer LFC. This is less than the 10.6% decrease in FAC thickness of the LFC reported by Kilic et al. [19] or the 9.2% decrease in FAC thickness reported by Coleman et al. [18] in the MFC from normal diurnal change. Mosher et al. [43] reported a maximum of 8% decrease in FAC thickness after 30 minutes of running.

Most MRI studies report cartilage volume change, rather than focal thickness data. Because the length and width of the cartilage plate are fixed, the volume change should be a function of thickness, making an indirect comparison of thickness to volume possible. Eckstein et al. [2] reported a 3.9% decrease in FAC volume at the MFC after 30 knee bends. Boocock et al. [22] reported a 5.3% decrease in FAC volume at the MFC after 5,000 running steps. Kersting et al. [21] showed a 3.1% decrease in FAC volume following a one-hour run. The decrease in FAC thickness of 6.94% after marathon running in the current study is similar to these earlier results and is less than the thickness changes reported for normal daily activity. Therefore, our ultrasonography results support evidence from previous MRI studies that marathon running does not induce greater knee cartilage deformation than other less strenuous activities [24-28].

The only significant area of FAC thickness change after the marathon was seen at the outer portion of the left LFC. This may be explained by the marathon course, which included a two-mile run along a beach, with the ocean to the right of the runners. The remainder of the course was on paved streets. The slight grade of the beach and right side of the road should have produced a mild valgus force at the left knee. This subtle difference could cause an asymmetrically greater force at the outermost portion of the left LFC. An equal increase in force and resulting deformation at the right medial knee might be expected but was not shown by the current study. The ankle, with greater inversion than eversion, may be better able to adapt to a surface that induces ankle inversion, e.g., the right ankle of a runner on the right side of a graded road. This was shown during a drop jump experiment, where landing from a height of 40 cm on a 3.6° laterally inclined surface that produced ankle eversion led to significantly higher valgus force at the knee [44]. Opposite results were not observed with a medial incline [44]. Fatigue may also affect ankle eversion, especially in novice runners [45]. This "angled course" hypothesis is speculative, however, because we do not know which side of the road the participants chose to run on or the potential grade of the beach portion of the course. However, this situation could be easily tested with a future study. Our unilateral findings highlight the importance of measuring and reporting effects for both legs in biomechanical studies.

The main limitation of this study is its observational nature and lack of a control group. The sample size was also small, which could explain why only one area showed significant thickness changes. Another important limitation is the lack of a more thorough ultrasound protocol for investigating the FAC. Li et al. [46] demonstrated with MRI that FAC thickness can vary among 12 points on each femoral condyle, separated by as little as 3 mm. Weight-bearing regions may become thinner with stress, while nonweight-bearing areas become thicker [17]. The current ultrasound protocol investigates only one plane on a very complex surface. It is possible that the areas of FAC exposed to the ultrasound beam at maximum knee flexion were not the areas undergoing the greatest deformation during the marathon. Other authors [32] have previously suggested the need for identifying which angle of knee flexion best reveals the weight-bearing portions of the femoral condyles. For future work, a protocol could be developed that included ultrasound imaging at multiple angles of knee flexion and measurement at multiple points on each condyle to better map the complex cartilage area visible by ultrasound imaging.

## Conclusions

Marathon running induced up to a 6.94% decrease in FAC thickness. This change was no greater than changes reported for other weight-bearing activities, including normal daily activities. Ultrasound imaging, because it is cost-effective and portable, may be useful for studying the physical characteristics of the articular cartilage, and the possibility of measuring FAC deformation might someday make it a more often-used tool to help understand the biomechanical forces exerted on the knee during activities such as marathon running.

## References

[1] Eckstein F, Tieschky M, Faber S, Englmeier KH, Reiser M (1999). Functional analysis of articular cartilage deformation, recovery, and fluid flow following dynamic exercise in vivo. Anat Embryol (Berl).

[2] Eckstein F, Lemberger B, Gratzke C, Hudelmaier M, Glaser C, Englmeier KH, Reiser M (2005). In vivo cartilage deformation after different types of activity and its dependence on physical training status. Ann Rheum Dis.

[3] Eckstein F, Lemberger B, Stammberger T, Englmeier KH, Reiser M (2000). Patellar cartilage deformation in vivo after static versus dynamic loading. J Biomech.

[4] Kessler MA, Glaser C, Tittel S, Reiser M, Imhoff AB (2006). Volume changes in the menisci and articular cartilage of runners: an in vivo investigation based on 3-D magnetic resonance imaging. Am J Sports Med.

[5] Kessler MA, Glaser C, Tittel S, Reiser M, Imhoff AB (2008). Recovery of the menisci and articular cartilage of runners after cessation of exercise: additional aspects of in vivo investigation based on 3-dimensional magnetic resonance imaging. Am J Sports Med.

[6] Van Ginckel A, Roosen P, Almqvist KF, Verstraete K, Witvrouw E (2011). Effects of in vivo exercise on ankle cartilage deformation and recovery in healthy volunteers: an experimental study. Osteoarthritis Cartilage.

[7] Hosseini A, Van de Velde SK, Kozanek M, Gill TJ, Grodzinsky AJ, Rubash HE, Li G (2010). In-vivo time-dependent articular cartilage contact behavior of the tibiofemoral joint. Osteoarthritis Cartilage.

[8] Bingham JT, Papannagari R, Van de Velde SK (2008). In vivo cartilage contact deformation in the healthy human tibiofemoral joint. Rheumatology (Oxford).

[9] Van Ginckel A, Witvrouw E (2013). Acute cartilage loading responses after an in vivo squatting exercise in people with doubtful to mild knee osteoarthritis: a case-control study. Phys Ther.

[10] Liu F, Kozanek M, Hosseini A, Van de Velde SK, Gill TJ, Rubash HE, Li G (2010). In vivo tibiofemoral cartilage deformation during the stance phase of gait. J Biomech.

[11] Birk GT, DeLee JC (2001). Osteochondral injuries. Clinical findings. Clin Sports Med.

[12] Curl WW, Krome J, Gordon ES, Rushing J, Smith BP, Poehling GG (1997). Cartilage injuries: a review of 31,516 knee arthroscopies. Arthroscopy.

[13] Widuchowski W, Widuchowski J, Trzaska T (2007). Articular cartilage defects: study of 25,124 knee arthroscopies. Knee.

[14] Brophy RH, Rodeo SA, Barnes RP, Powell JW, Warren RF (2009). Knee articular cartilage injuries in the National Football League: epidemiology and treatment approach by team physicians. J Knee Surg.

[15] Murphy L, Helmick CG (2012). The impact of osteoarthritis in the United States: a population-health perspective. Am J Nurs.

[16] Hada S, Kaneko H, Sadatsuki R (2014). The degeneration and destruction of femoral articular cartilage shows a greater degree of deterioration than that of the tibial and patellar articular cartilage in early stage knee osteoarthritis: a cross-sectional study. Osteoarthritis Cartilage.

[17] Waterton JC, Solloway S, Foster JE (2000). Diurnal variation in the femoral articular cartilage of the knee in young adult humans. Magn Reson Med.

[18] Coleman JL, Widmyer MR, Leddy HA (2013). Diurnal variations in articular cartilage thickness and strain in the human knee. J Biomech.

[19] Kilic G, Kilic E, Akgul O, Ozgocmen S (2015). Ultrasonographic assessment of diurnal variation in the femoral condylar cartilage thickness in healthy young adults. Am J Phys Med Rehabil.

[20] van den Bogert AJ, Read L, Nigg BM (1999). An analysis of hip joint loading during walking, running, and skiing. Med Sci Sports Exerc.

[21] Kersting UG, Stubendorff JJ, Schmidt MC, Brüggemann GP (2005). Changes in knee cartilage volume and serum COMP concentration after running exercise. Osteoarthritis Cartilage.

[22] Boocock M, McNair P, Cicuttini F, Stuart A, Sinclair T (2009). The short-term effects of running on the deformation of knee articular cartilage and its relationship to biomechanical loads at the knee. Osteoarthritis Cartilage.

[23] Willick SE, Hansen PA (2010). Running and osteoarthritis. Clin Sports Med.

[24] Krampla W, Mayrhofer R, Malcher J, Kristen KH, Urban M, Hruby W (2001). MR imaging of the knee in marathon runners before and after competition. Skeletal Radiol.

[25] Schueller-Weidekamm C, Schueller G, Uffmann M, Bader TR (2006). Does marathon running cause acute lesions of the knee? Evaluation with magnetic resonance imaging. Eur Radiol.

[26] Krampla WW, Newrkla SP, Kroener AH, Hruby WF (2008). Changes on magnetic resonance tomography in the knee joints of marathon runners: a 10-year longitudinal study. Skeletal Radiol.

[27] Hohmann E, Wörtler K, Imhoff AB (2004). MR imaging of the hip and knee before and after marathon running. Am J Sports Med.

[28] Luke AC, Stehling C, Stahl R (2010). High-field magnetic resonance imaging assessment of articular cartilage before and after marathon running: does long-distance running lead to cartilage damage?. Am J Sports Med.

[29] Aisen AM, McCune WJ, MacGuire A, Carson PL, Silver TM, Jafri SZ, Martel W (1984). Sonographic evaluation of the cartilage of the knee. Radiology.

[30] McCune WJ, Dedrick DK, Aisen AM, MacGuire A (1990). Sonographic evaluation of osteoarthritic femoral condylar cartilage. Correlation with operative findings. Clin Orthop Relat Res.

[31] Möller I, Bong D, Naredo E, Filippucci E, Carrasco I, Moragues C, Iagnocco A (2008). Ultrasound in the study and monitoring of osteoarthritis. Osteoarthritis Cartilage.

[32] Lee CL, Huang MH, Chai CY, Chen CH, Su JY, Tien YC (2008). The validity of in vivo ultrasonographic grading of osteoarthritic femoral condylar cartilage: a comparison with in vitro ultrasonographic and histologic gradings. Osteoarthritis Cartilage.

[33] Tarhan S, Unlu Z (2003). Magnetic resonance imaging and ultrasonographic evaluation of the patients with knee osteoarthritis: a comparative study. Clin Rheumatol.

[34] Naredo E, Acebes C, Möller I (2009). Ultrasound validity in the measurement of knee cartilage thickness. Ann Rheum Dis.

[35] Castriota-Scanderbeg A, De Micheli V, Scarale MG, Bonetti MG, Cammisa M (1996). Precision of sonographic measurement of articular cartilage: inter- and intraobserver analysis. Skeletal Radiol.

[36] Mathiesen O, Konradsen L, Torp-Pedersen S, Jørgensen U (2004). Ultrasonography and articular cartilage defects in the knee: an in vitro evaluation of the accuracy of cartilage thickness and defect size assessment. Knee Surg Sports Traumatol Arthrosc.

[37] Myers SL, Dines K, Brandt DA, Brandt KD, Albrecht ME (1995). Experimental assessment by high frequency ultrasound of articular cartilage thickness and osteoarthritic changes. J Rheumatol.

[38] Spannow AH, Pfeiffer-Jensen M, Andersen NT, Stenbøg E, Herlin T (2009). Inter -and intraobserver variation of ultrasonographic cartilage thickness assessments in small and large joints in healthy children. Pediatr Rheumatol Online J.

[39] Iagnocco A, Coari G, Zoppini A (1992). Sonographic evaluation of femoral condylar cartilage in osteoarthritis and rheumatoid arthritis. Scand J Rheumatol.

[40] Eryılmaz ÖG, Kara M, Tiftik T (2012). Ultrasonographic measurement of the femoral cartilage thickness in patients with polycystic ovary syndrome. Fertil Steril.

[41] Kaya A, Kara M, Tiftik T, Tezcan ME, Öztürk MA, Akıncı A, Özçakar L (2013). Ultrasonographic evaluation of the femoral cartilage thickness in patients with systemic lupus erythematosus. Rheumatol Int.

[42] Kilic G, Kilic E, Akgul O, Ozgocmen S (2014). Decreased femoral cartilage thickness in patients with systemic sclerosis. Am J Med Sci.

[43] Mosher TJ, Liu Y, Torok CM (2010). Functional cartilage MRI T2 mapping: evaluating the effect of age and training on knee cartilage response to running. Osteoarthritis Cartilage.

[44] Hagins M, Pappas E, Kremenic I, Orishimo KF, Rundle A (2007). The effect of an inclined landing surface on biomechanical variables during a jumping task. Clin Biomech (Bristol, Avon).

[45] Koblbauer IF, van Schooten KS, Verhagen EA, van Dieën JH (2014). Kinematic changes during running-induced fatigue and relations with core endurance in novice runners. J Sci Med Sport.

[46] Li G, Park SE, DeFrate LE, Schutzer ME, Ji L, Gill TJ, Rubash HE (2005). The cartilage thickness distribution in the tibiofemoral joint and its correlation with cartilage-to-cartilage contact. Clin Biomech (Bristol, Avon).

